# An effective numerical method to solve a class of nonlinear singular boundary value problems using improved differential transform method

**DOI:** 10.1186/s40064-016-2753-9

**Published:** 2016-07-12

**Authors:** Lie-jun Xie, Cai-lian Zhou, Song Xu

**Affiliations:** Department of Mathematics, Ningbo University, Fenghua Road 818, Jiangbei District, Ningbo City, 315211 Zhejiang Province People’s Republic of China

**Keywords:** Singular boundary value problem, Differential transform method, Adomian polynomials, Improved differential transform method, Approximate series solutions

## Abstract

In this work, an effective numerical method is developed to solve a class of singular boundary value problems arising in various physical models by using the improved differential transform method (IDTM). The IDTM applies the Adomian polynomials to handle the differential transforms of the nonlinearities arising in the given differential equation. The relation between the Adomian polynomials of those nonlinear functions and the coefficients of unknown truncated series solution is given by a simple formula, through which one can easily deduce the approximate solution which takes the form of a convergent series. An upper bound for the estimation of approximate error is presented. Several physical problems are discussed as illustrative examples to testify the validity and applicability of the proposed method. Comparisons are made between the present method and the other existing methods.

## Background

Singular boundary value problems (SBVPs) is an important class of boundary value problems, and arises frequently in the modeling of many actual problems related to physics and engineering areas such as in the study of electro hydrodynamics, theory of thermal explosions, boundary layer theory, the study of astrophysics, three layer beam, electromagnetic waves or gravity driven flows, inelastic flows, the theory of elastic stability and so on. In general, SBVPs is difficult to solve analytically. Therefore, various numerical techniques have been proposed to treat it by many researchers. However, the solution of SBVPs is numerically challenging due to the singularity behavior at the origin.

In this work, we are interested again in the following SBVPs arising frequently in applied science and engineering:

1 $$u^{\prime\prime}(x)+\frac{\alpha }{x}u^{\prime}(x)=f(x,u), \quad 0<x\le 1, \quad \alpha \ge 1,$$

subject to the boundary value conditions

2 $$u^{\prime}(0)=0$$

and

3 $$au(1)+bu^{\prime}(1)=c,$$

where *a*, *b* and *c* are any finite real constants. If $\alpha =1$, (1) becomes a cylindrical problem, and it becomes a spherical problem when $\alpha =2$. It is assumed that *f*(*x*, *u*) is continuous, $\frac{\partial f}{\partial u}$ exists and is continuous and $\frac{\partial f}{\partial u}\ge 0$ for any $0<x\le 1$ such that Eq. (1) has a unique solution (Russell and Shampine 1975).

The SBVPs (1–3) with different $\alpha$ arise in the study of various scientific problems for certain linear or nonlinear functions *f*(*x*, *u*). The common cases related to the actual problems are summarized as follows. The first case for $\alpha =2$ and

4 $$f(x,u)=f(u)=\frac{\delta u(x)}{u(x)+\mu }$$

emerges from the modeling of steady state oxygen diffusion in a spherical cell with Michaelis–Menten uptake kinetics (Lin 1976; McElwain 1978). In this case, *u*(*x*) represents the oxygen tension; $\delta$ and $\mu$ are positive constants involving the reaction rate and the Michaelis constant. Hiltmann and Lory (1983) proposed the existence and uniqueness of the solution for $b=1$ and $a=c$. Analytical bounding functions were given in Anderson and Arthurs (1985). The numerical methods to solve the SBVPs for this case have attracted a reasonable amount of research works, such as the finite difference method (FDM) (Pandey 1997), the cubic spline method (CSM) (Rashidinia et al. 2007; Ravi and Bhattacharya 2006), the Sinc-Galerkin method (SGM) (Babolian et al. 2015), the Adomian decomposition method (ADM) and its modified methods (Khuri and Sayfy 2010; Wazwaz et al. 2013; Singh and Kumar 2014), the variational iteration method (VIM) (Ravi and Aruna 2010; Wazwaz 2011), the series expansion technique (SEM) (Turkyilmazoglu 2013) and the B-spline method (BSM) (Çağlar et al. 2009).

The second case arises in the study of the distribution of heat sources in the human head (Flesch 1975; Gray 1980; Duggan and Goodman 1986), in which $\alpha =2$ and

5 $$f(x,u)=f(u)=-le^{-lku(x)},\quad l>0,\; k>0.$$

In Duggan and Goodman (1986), point-wise bounds and uniqueness results were presented for the SBVPs with the nonlinear function *f*(*x*, *u*) of the forms given by (4) and (5). Quite a little amount of works by using different approaches, including the FDM (Pandey 1997), the CSM (Rashidinia et al. 2007; Ravi and Bhattacharya 2006) and the SGM (Babolian et al. 2015), have been proposed to obtain the approximate solutions of this case.

The third important case of physical significance is when $\alpha =1, 2$ and

6 $$f(x,u)=f(u)=\nu e^{u(x)},$$

which arises in studying the theory of thermal explosions (Khuri and Sayfy 2010; Kumar and Singh 2010; Chang 2014) and the electric double layer in a salt-free solution (Chang 2012). A variety of numerical methods have been applied to handle such SBVPs, for example, the fourth order finite difference method (FFDM) (Chawla et al. 1988), the modified Adomian decomposition method (Khuri and Sayfy 2010; Singh and Kumar 2014; Kumar and Singh 2010), the Taylor series method (TSM) (Chang 2014) and the BSM (Çağlar et al. 2009).

Besides, Chandrasekhar (1939) derived another case for $\alpha =2, b=0$ and

7 $$f(x,u)=f(u)=-u^{\gamma }(x),$$

which $\gamma$ is a physical constant. This case is in connection with the equilibrium of thermal gas thermal (Ames 1968). The numerical solution of this kind of equation for $\gamma =5$ was considered by using various methods, such as the FFDM (Chawla et al. 1988), the VIM (Ravi and Aruna 2010), the SEM (Turkyilmazoglu 2013) and the modified Adomian decomposition method (Singh and Kumar 2014).

All the aforementioned methods can yield a satisfied result. However, each of these methods has its own weaknesses. For example, the VIM (Ravi and Aruna 2010; Wazwaz 2011) has an inherent inaccuracy in identifying the Lagrange multiplier, and fails to solve the equation when the nonlinear function *f*(*x*, *u*) is of the forms (5) and (6). Those methods such as the FDM (Pandey 1997; Chawla et al. 1988), the SEM (Turkyilmazoglu 2013), the SGM (Babolian et al. 2015) and the spline method (Rashidinia et al. 2007; Ravi and Bhattacharya 2006; Çağlar et al. 2009) require a tedious process and huge volume of computations in dealing with the linearization or discretization of variables. The ADM (Wazwaz et al. 2013) needs to obtain the corresponding Volterra integral form of the given equation, via which one can overcome the difficulty of singular behavior at $x=0$. The modified ADM (Khuri and Sayfy 2010; Kumar and Singh 2010) needs to introduce a twofold indefinite integral operator to give better and accurate results; moreover, the success of method in (Singh and Kumar 2014) relies on constructing Green’s function before establishing the recursive relation for applying the ADM to derive the solution components. All those manners are at the expense of computation budgets. Besides, none of above methods is applied to handle the equations with all forms of nonlinearities (4–7).

In recent years, a lot of attentions have been devoted to the applications of differential transform method (DTM) and its modifications. The DTM proposed by Pukhov (1980, 1982, 1986) at the beginning of 1980s. However, his work passed unnoticed. In 1986, Zhou (1986) reintroduced the DTM to solve the linear and nonlinear equations in electrical circuit problems. The DTM is a semi-numerical-analytic method that generates a Taylor series solution in the different manner. In the past forty years, the DTM has been successfully applied to solve a wide variety of functional equations; see Xie et al. (2016) and the references therein.

Although being powerful, there still exist some difficulties in solving various of equations by the classical DTM. Some researchers have devoted to deal with these obstacles so as to extend the applications of the DTM. For example, in view of the DTM numerical solution cannot exhibit the real behaviors of the problem, Odibat et al. (2010) proposed a multi-step DTM to accelerate the convergence of the series solution over a large region and applied successfully to handle the Lotka-Volterra, Chen and Lorenz systems. In Gökdoğan et al. (2012), Momani and Ertürk (2008) suggested an alternative scheme to overcome the difficulty of capturing the periodic behavior of the solution by combining the DTM, Laplace transform and Padé approximants. Another difficulty is to compute the differential transforms of the nonlinear components in a simple and effective way. By using the traditional approach of the DTM, the computational difficulties will inevitably arise in determining the transformed function of an infinity series. Compared to the traditional method, Chang and Chang (2008) proposed a relatively effective algorithm for calculating the differential transform through a derived recursive relation. Yet, by using their method, it is inevitable to increase the computational budget, especially in dealing with those differential equations which have two or more nonlinear terms being investigated. Recently, the authors Elsaid (2012), Fatoorehchi and Abolghasemi (2013) disclosed the relation between the Adomian polynomials and the differential transform of nonlinearities, and developed an inspiring approach to handle the nonlinear functions in the given functional equation. Meanwhile, the problem of tedious calculations in dealing with nonlinear problems by using the ADM has also been improved considerably by Duan (2010a, b, 2011). All of these effective works make it possible to broaden the applicability and popularity of the DTM considerably.

The aim of this work is to develop an efficient approach to solve the SBVPs (1–3) with those nonlinear terms (4–7). This scheme is mainly based on the improved differential transform method (IDTM), which is the improved version of the classical DTM by using the Adomian polynomials to handle the differential transforms of those nonlinear functions (4–7). No specific technique is required in dealing with the singular behavior at the origin. Meanwhile, unlike some existing approaches, the proposed method tackles the problem in a straightforward manner without any discretization, linearization or perturbation. The numerical solution obtained by the proposed method takes the form of a convergent series with those easily computable coefficients through the Adomian polynomials of those nonlinear functions as the forms of (4–7).

The rest of the paper is organized as follows. In the next section, the concepts of DTM and Adomian polynomials are introduced. Algorithm for solving the problem (1–3) and an upper bound for the estimation of approximate error are presented in Sect. 3. Sect.4 shows some numerical examples to testify the validity and applicability of the proposed method. In Sect. 5, we end this paper with a brief conclusion.

## Adomian polynomial and differential transform

### Adomian polynomial

In the Adomian decomposition method (ADM), a key notion is the Adomian polynomials, which are tailored to the particular nonlinearity to easily and systematically solve nonlinear differential equations. The interested readers are referred to Adomian (1990, 1994) for the details of the ADM.

For the applications of decomposition method, the solution of the given equation in a series form is usually expressed by

8 $$u= \sum _{m=0}^{\infty } u_m,$$

and the infinite series of polynomials

9 $$f(u)=f \left( \sum _{m=0}^{\infty } u_m \right) =\sum _{m=0}^{\infty } A_{m}$$

for the nonlinear term *f*(*u*), where $A_{m}$ is called the Adomian polynomials, and depends on the solution components $u_0, u_1, \ldots , u_m$. The traditional algorithm for evaluating the Adomoan polynomials $A_n$ was first provided in Adomian and Rach (1983) by the formula

10 $$\left. A_{n}= \frac{1}{n!} \frac{\hbox {d}^n}{\hbox {d} \lambda ^n} f \left( \sum _{m=0}^{\infty } u_m \lambda ^m \right) \right| _{\lambda =0}.$$

A large amount of works (Duan 2010b, b, 2011; Adomian and Rach 1983; Rach 2008, 1984; Wazwaz 2000; Abbaoui et al. 1995; Abdelwahid 2003; Azreg-AÏnou 2009) have been applied to give the more effective computational method for the Adomian polynomials. For fast computer generation, we favor Duan’s Corollary 3 algorithm (Duan 2011) among all of these methods, as it merely involves the analytic operations of addition and multiplication without the differentiation operator, which is eminently convenient for symbolic implementation by computer algebraic systems such as Maple and Mathematics. The method to generate the Adomian polynomials in Duan (2011) is described as follows:

11 $$\begin{array}{ll} C_{n}^1= u_n, \quad n \ge 1,\\ C_{n}^k= \frac{1}{n} \sum _{j=0}^{n-k} (j+1)u_{j+1}C_{n-1-j}^{k-1}, \quad 2\le k \le n, \end{array}$$

such that

12 $$\begin{array}{ll} y A_0= f(u_0),\\ \displaystyle A_n= \sum _{k=1}^{n} C_{n}^{k} f^{(k)}(u_0), \quad n \ge 1. \end{array}$$

It is worth mentioning that Duan’s algorithm involving (11) and (12) has been testified to be one of the fastest subroutines on record (Duan 2011), including the fast generation method given by Adomian and Rach (1983).

### Differential transform

The differential transform of the $k{th}$ differentiable function *u*(*x*) at $x=0$ is defined by

13 $$U(k)=\frac{1}{k!} \left[ \frac{\hbox {d}^k u(x)}{\hbox {d}x^k} \right] _{x=0},$$

and the differential inverse transform of *U*(*k*) is described as

14 $$u(x)=\sum _{k=0}^{\infty }U(k) x^k,$$

where *u*(*x*) is the original function and *U*(*k*) is the transformed function.

For the practical applications, the function *u*(*x*) is expressed by a truncated series and Eq. (14) can be written as

15 $$u(x)\approx u_N(x)=\sum _{k=0}^{N}U(k) x^k.$$

It is not difficult to deduce the transformed functions of the fundamental operations listed in Table 1.

**Table 1:** The fundamental operations of the DTM

Original function	Transformed function
$w(x)=\alpha u(x)\pm \beta v(x)$	$W(k)=\alpha U(k)\pm \beta V(k)$
$w(x)=u(x)v(x)$	$W(k)=\sum _{m=0}^k U(m)V(k-m)$
$w(x)=\hbox {d}^m u(x)/\hbox {d}x^m$	$W(k)= \frac{(k+m)!}{k!}U(k+m)$
$w(x)=x^m$	$$W(k)= \delta (k-m)=\left\{ \begin{array}{ll} 1, \quad \hbox {if}\; k=m,\\ 0, \quad \hbox {if} \; k\ne m. \end{array} \right.$$
$w(x)=\exp (x)$	$W(k)= 1/k!$
$w(x)=\sin (\alpha x+\beta )$	$W(k)= \alpha ^k/k! \sin (k \pi /2+\beta )$
$w(x)=\cos (\alpha x+\beta )$	$W(k)= \alpha ^k/k! \cos (k \pi /2+\beta )$

Note that $\alpha , \beta$ are constants and *m* is a nonnegative integer

## Method of solution of SBVPs (1–3)

We want to find the approximate solution of the problem (1–3) with the type:

16 $$u_N(x)=\sum _{k=0}^{N}U(k) x^k,$$

where the coefficients $U(0),U(1),\ldots ,U(N)$ are determined using the following steps:

- According to the definition (13) of the differential transform and the boundary value condition (2), we have
  17 $$U(1)=0.$$
  Suppose that
  18 $$U(0)=\beta,$$
  where $\beta$ is a real parameter to be determined.
- Multiplying both sides of Eq. (1) by variable *x*, we have
  19 $$xu^{\prime}(x)+\alpha u^{\prime}(x)=xf(x,u).$$
  Applying the differential transform (13) to Eq. (19), we get the following recurrence relation:
  20 $$U(k+1)=\frac{F(k-1)}{(k+1)(k+\alpha )}, \quad k=1,2,\ldots ,N-1,$$
  where *F*(*k*) is the differential transform of the nonlinear function $f(x,u)=f(u)$.
- Using Lemma 3.1 in Fatoorehchi and Abolghasemi (2013), we compute *F*(*k*) through the Adomian polynomials $A_k$:
  21 $$F(k)=A_k, \quad k=0,1,2,\ldots ,N.$$

### *Remark 1*

Lemma 3.1 in Fatoorehchi and Abolghasemi (2013) indicates that the differential transforms and the Adomian polynomials of nonlinear functions have the same mathematical structure such that we can derive the differential transforms of any nonlinear functions by merely calculating the relevant Adomian polynomials but with constants instead of variable components.

### *Remark 2*

As mentioned before, we use Duan’s Corollary 3 algorithm (Duan 2011) (11–12) to generate the Adomian polynomials.

- Substituting (21) into (20), and then combining the relations (16–18), we obtain the truncated series solution of the problem (1–3) as follows:
  22 $$u_N(x)=\beta +\sum _{k=1}^{N-1}\frac{A_{k-1}}{(k+1)(k+\alpha )} x^{k+1}.$$
- Imposing the truncated series solution (22) on the boundary condition (3), we obtain a nonlinear algebraic equation with unknown parameter $\beta$:
  23 $$g(\beta )=0.$$
  Solving Eq. (23), and substituting the value of $\beta$ into (22), we obtain the final result.

An upper bound for the estimation of approximate error is presented in the following lemma.

### **Lemma 1**

*Suppose that*$u(x) \in C^{N+1}[0,1]$*is the exact solution of the problem* (1–3), $u_N(x)=\sum _{k=0}^{N}U(k)x^k$*is the truncated series solution with degreeN*, *it holds that*

24 $$||u(x)-u_N(x)||_{\infty } \le \frac{M}{(N+1)!}+\max \limits _{0\le k\le N} \left| c_k \right| ,$$

*where*$$M=\max \limits _{0\le x\le 1} | u^{(N+1)}(x)|, c_k= \frac{u^{(k)}(0)}{k!}-U(k)$$.

### *Proof*

Obviously, we have

25 $$||u(x)-u_N(x)||_{\infty } \le ||u(x)- \widetilde{u}_N(x)||_{\infty } +||\widetilde{u}_N(x)-u_N(x)||_{\infty },$$

where $\widetilde{u}_N(x)=\sum _{k=0}^{N} \frac{u^{(k)}(0)}{k!}x^k$ is the Taylor polynomial of the unknown function *u*(*x*) at $x=0$.

Since $u(x) \in C^{N+1}[0,1]$, it follows that

$$u(x)=\widetilde{u}_N(x) + R_N(x)=\widetilde{u}_N(x) + \frac{u^{(N+1)}(\xi )}{(N+1)!}x^{N+1}, \quad \xi \in (0,1),$$

where $R_N(x)$ is the remainder of Taylor polynomial $\widetilde{u}_N(x)$. Therefore

26 $$\left| u(x)-\widetilde{u}_N(x)\right| =\left| R_N(x) \right| =\left| \frac{u^{(N+1)}(\xi )}{(N+1)!}x^{N+1} \right| \le \frac{1}{(N+1)!} \max \limits _{0\le x\le 1} \left| u^{(N+1)}(x) \right|.$$

Let

$${\mathbf{C}}= (c_0,c_1,\ldots ,c_N), \quad {\mathbf{\Theta}}=(x^0,x^1,\ldots ,x^N)^T,$$

where

$$c_k= \frac{u^{(k)}(0)}{k!}-U(k), \quad k=0,1,\ldots ,N.$$

We then have

27 $$\left| \widetilde{u}_N(x)- u_N(x) \right| = \left| \sum _{k=0}^{N} \left( \frac{u^{(k)}(0)}{k!}-U(k) \right) x^k \right| =\left| \mathbf{C} \cdot \mathbf{\Theta } \right| \le ||\mathbf{C}||_{\infty } \cdot ||\mathbf{\Theta }||_{\infty }$$

Combining the relations (25–27), it follows that

28 $$\begin{array}{ll} \displaystyle ||u(x)-u_N(x)||_{\infty } \le \frac{1}{(N+1)!} \max \limits _{0\le x\le 1} \left| u^{(N+1)}(x) \right| + ||\mathbf{C}||_{\infty } \cdot ||\mathbf{\Theta }||_{\infty }\\ \le \frac{M}{(N+1)!}+ \max \limits _{0\le k\le N} \left| c_k \right| . \end{array}$$

Thus, the proof is completed. $\square$

## Numerical examples

In this section, based on the discussion in Sect. 3, we report numerical tests of five classical examples discussed frequently to testify the validity and applicability of the proposed method. All the numerical computations were performed using Maple and Matlab on personal computer. For comparison, we computed the absolute error defined by

29 $$\hbox {E}_N(x)=\left| u(x)-u_N(x)\right|$$

and the maximal absolute error by

30 $$\hbox {ME}_N=\max \limits _{0 \le x \le 1} \left| u(x)-u_N(x)\right| ,$$

where *u*(*x*) is the exact solution and $u_N(x)$ is the truncated series solution with degree *N*.

### *Example 1*

Consider the following nonlinear SBVP in the study of isothermal gas sphere (Singh and Kumar 2014; Ravi and Aruna 2010; Chawla et al. 1988):

31 $$u^{\prime\prime}(x)+\frac{2}{x} u^{\prime}(x)=-u^5(x),$$

subject to the boundary conditions

32 $$u^{\prime}(0)=0, \quad u(1)=\frac{\sqrt{3}}{2}.$$

The exact solution of this problem is given by $u(x)=\sqrt{\frac{3}{3+x^2}}$. It is also known as the Emden-Fowler equation of the first kind. In what follows, we shall solve it with the proposed algorithm.

Firstly, we set

$$U(0)=\beta , \quad U(1)=0.$$

The Adomian polynomials of nonlinear term $f(x,u)=-u^5(x)$ in this problem are computed as

$$\begin{aligned} A_0&= -U^5(0),\\ A_1&= -5U^4(0)U(1) ,\\ A_2&= -10U^3(0)U^2(1)-5U^4(0)U(2),\\ A_3&= -10U^2(0)U^3(1)-20U^3(0)U(1)U(2)-5U^4(0)U(3),\\&\vdots&\end{aligned}$$

Furthermore, according to the relations (20) and (21), we obtain the differential transforms *U*(*k*) of the unknown function *u*(*x*)

$$\begin{aligned} U(2)&= \frac{1}{2\cdot 3} A_0=-\frac{1}{6}\beta ^5, \\ U(4)&= \frac{1}{4\cdot 5} A_3=\frac{1}{24}\beta ^9,\\ U(6)&= \frac{1}{6\cdot 7} A_5=-\frac{5}{432}\beta ^{13},\\&\vdots&\\ U(k)&=0, \quad \hbox {if}\; k \quad \hbox {is odd and } \; k \ge 3. \end{aligned}$$

By using Eq. (22), we obtain the truncated series solution for $N=10$ as follows:

33 $$u_{10}(x)=\beta -\frac{1}{6}\beta ^5x^2+\frac{1}{24}\beta ^9x^4-\frac{5}{432}\beta ^{13}x^6+\frac{35}{10368}\beta ^{17}x^8-\frac{7}{6912}\beta ^{21}x^{10}.$$

Secondly, imposing the truncated series solution (33) on the boundary conditions $u(1)=\sqrt{3}/2$, we get a nonlinear algebraic equation. By solving it, the unknown parameter $\beta$ is computed as

34 $$\beta =1.000553890.$$

Finally, substituting (34) into (33), we get the approximate solution with degree 10

$$\begin{aligned} u_{10}(x)&= 1.000553890-0.1671287533x^2+(0.4187483621e-1)x^4-\\&(0.1165769154e-1)x^6+(0.3407699551e-2)x^8-\\&(0.1024576736e-2)x^{10}. \end{aligned}$$

In Table 2, we compare the absolute errors (29) of numerical results obtained by the present method, the VIM (Ravi and Aruna 2010) and the modified ADM using Green functions (GIDM) (Singh and Kumar 2014) for $N=12$. Table 3 lists the theoretical estimate errors (24) and the maximal absolute errors (30) of the approximate solutions for changing approximation levels, and shows a comparison of the maximal absolute errors with the GIDM (Singh and Kumar 2014) and the FFDM (Chawla et al. 1988). We can see from Table 3 that the accuracy of our computational results is getting better as the approximation level is increasing. Moreover, our numerical solution $u_{10}(x)$ has an accuracy of O($10^{-4}$), whereas the GIDM (Singh and Kumar 2014) needs to employ 14 terms to archive this goal as shown in Table 1 of Singh and Kumar (2014); numerical solution with even 64 terms obtained by the FFDM (Chawla et al. 1988) still hovers at this level. In summary, Tables 2 and 3 indicate that the results of our proposed method have higher accuracy than of the GIDM (Singh and Kumar 2014), the FFDM (Chawla et al. 1988) and the VIM (Ravi and Aruna 2010).

**Table 2:** Comparison of the absolute error $\hbox {E}_{12}(x)$ for Example 1

*x*	GIDM (Singh and Kumar 2014)	VIM (Ravi and Aruna 2010)	Present method
0.0	3.1880e−03	6.3220e−03	1.6776e−04
0.1	3.1209e−03	6.2702e−03	1.6637e−04
0.2	2.9269e−03	6.1173e−03	1.6227e−04
0.3	2.6263e−03	5.8687e−03	1.5568e−04
0.4	2.2489e−03	5.5281e−03	1.4691e−04
0.5	1.8284e−03	5.0903e−03	1.3639e−04
0.6	1.3978e−03	4.5347e−03	1.2450e−04
0.7	9.8413e−04	3.8201e−03	1.1132e−04
0.8	6.0707e−04	2.8837e−03	9.5269e−05
0.9	2.7774e−04	1.6426e−03	6.8180e−05
1.0	3.52e−08	1.00e−10	0

**Table 3:** The theoretical estimate errors $\hbox {TE}_N$ and comparison of the maximal absolute errors $\hbox {ME}_N$ of present method and of other methods for Example 1

*N*	$\hbox {TE}_N$	$\hbox {ME}_N$	*N*	$\hbox {TE}_N$	$\hbox {ME}_N$	*N*	in Singh and Kumar (2014)	*N*	in Chawla et al. (1988)
6	1.83e−02	6.80e−03	12	4.7721e−04	1.6776e-04	12	1.3978e−03	16	3.64e−04
8	5.10e−03	1.70e−03	16	4.6453e−05	1.6521e-05	16	2.4654e−04	32	2.49e−04
10	1.5666e−03	5.5389e−04	20	4.6453e−06	1.6614e-06	20	4.8643e−05	64	1.60e−04

### *Example 2*

Consider the following nonlinear SBVP (Khuri and Sayfy 2010; Singh and Kumar 2014; Çağlar et al. 2009; Chawla et al. 1988):

35 $$u^{\prime\prime}(x)+\frac{1}{x} u^{\prime}(x)=-e^{u(x)},$$

subject to the boundary conditions

36 $$u^{\prime}(0)=0, \quad u(1)=0.$$

The exact solution is given by $u(x)=2 \ln \frac{C+1}{Cx^2+1}$, where $C=3-2\sqrt{2}$.

The Adomian polynomials of nonlinear term $f(x,u)=-e^{u(x)}$ in this problem are computed as

$$\begin{aligned} A_0&= -e^{U(0)},\\ A_1&= -U(1)e^{U(0)},\\ A_2&= -U(2)e^{U(0)}-\frac{1}{2}U^2(1)e^{U(0)},\\ A_3&= -U(3)e^{U(0)}-U(1)U(2)e^{U(0)}-\frac{1}{6}U^3(1)e^{U(0)},\\&\vdots&\end{aligned}$$

A comparison of the absolute errors (29) of the numerical solutions for $N=10,\;20,\;40$ obtained by the present method and the modified decomposition method (BSDM) (Khuri and Sayfy 2010) is described in Table 4. Table 5 lists the maximal absolute errors (30) of those numerical results derived from the proposed method, the BSM (Çağlar et al. 2009) and the FFDM (Chawla et al. 1988). And also, we list the theoretical estimate errors (24) in Table 5 for comparison. It can be seen from Tables 4 and 5 that one can obtain the better approximate solution by using the present method compared to the other mentioned methods, even if we take the relative smaller *N*. Moreover, the theoretical estimate errors, the absolute errors and the maximal absolute errors all decrease as the increase of *N*. Therefore, evaluation of more components of the numerical solution will reasonably improve the accuracy.

**Table 4:** Comparison of the absolute errors $\hbox {E}_N(x)$ for Example 2

*x*	BSDM Khuri and Sayfy (2010)			Present method		
	$\hbox {E}_{10}(x)$	$\hbox {E}_{20}(x)$	$\hbox {E}_{40}(x)$	$\hbox {E}_{10}(x)$	$\hbox {E}_{20}(x)$	$\hbox {E}_{40}(x)$
0.0	1.05e−05	1.05e−05	1.05e−05	1.05e−05	2.2e−09	1.4e−09
0.1	1.05e−05	1.05e−05	1.05e−05	1.05e−05	1.2e−09	4.0e−10
0.2	1.03e−05	1.03e−05	1.03e−05	1.03e−05	1.4e−09	6.0e−10
0.3	1.02e−05	1.02e−05	1.02e−05	1.02e−05	1.4e−09	6.0e−10
0.4	9.93e−06	9.93e−06	9.93e−06	9.93e−06	1.5e−09	8.0e−10
0.5	9.62e−06	9.62e−06	9.62e−06	9.62e−06	2.6e−09	1.8e−09
0.6	2.73e−06	6.07e−06	6.93e−06	9.25e−06	1.9e−09	1.2e−09
0.7	6.67e−07	3.65e−06	4.75e−06	8.75e−06	1.4e−09	7.0e−10
0.8	1.58e−06	2.02e−06	2.93e−06	7.88e−06	9.0e−10	3.0e−10
0.9	1.08e−06	8.76e−07	1.37e−06	5.78e−06	5.5e−10	1.1e−09
1.0	0	0	0	1.10e−10	2.74e−11	3.6e−11

**Table 5:** The theoretical estimate errors $\hbox {TE}_N$ and comparison of the maximal absolute errors $\hbox {ME}_N$ of present method and of other methods for Example 2

*N*	$\hbox {TE}_N$	$\hbox {ME}_N$	*N*	$\hbox {TE}_N$	$\hbox {ME}_N$	*N*	in Çağlar et al. (2009)	*N*	in Chawla et al. (1988)
10	6.9957e−05	1.0488e−05	16	2.2413e−07	3.5041e−08	20	3.1607e−05	16	2.52e−03
12	1.0042e−05	1.5380e−06	18	3.2730e−08	5.4593e−09	40	7.8742e−06	32	1.83e−04
14	1.4795e−06	2.3036e−07	20	6.6210e−09	8.4075e−10	60	3.5011e−06	64	1.28e−05

### *Example 3*

Consider the following nonlinear SBVP in the study of steady-state oxygen diffusion in a spherical cell (Babolian et al. 2015; Khuri and Sayfy 2010; Wazwaz 2011; Çağlar et al. 2009):

37 $$u^{\prime\prime}(x)+\frac{\alpha }{x} u^{\prime}(x)=\frac{\delta u(x)}{u(x)+\mu }, \quad \delta>0, \quad \mu >0,$$

subject to the boundary conditions

38 $$u^{\prime}(0)=0, \quad 5u(1)+u^{\prime}(1)=5,$$

where $\delta$ and $\mu$ are often taken as 0.76129 and 0.03119, respectively. We take the value of $\alpha$ as 1, 2 and 3.

The Adomian polynomials of nonlinear term $f(x,u)=\frac{\delta u(x)}{u(x)+\mu }$ in this problem are computes as

$$\begin{aligned} A_0&= \frac{\delta }{U(0)+\mu }U(0),\\ A_1&= \frac{\delta \mu }{(U(0)+\mu )^2}U(1) ,\\ A_2&= \frac{\delta \mu }{(U(0)+\mu )^2} U(2) - \frac{ \delta \mu }{(U(0)+\mu )^3} U^2(1),\\ A_3&= \frac{\delta \mu }{(U(0)+\mu )^2} U(3) - \frac{2\delta \mu }{(U(0)+\mu )^3}U(1)U(2)+\frac{ \delta \mu }{(U(0)+\mu )^4} U^3(1),\\&\vdots&\end{aligned}$$

Proceeding as before, we compute the approximate solution $u_{12,2}(x)$ for $N=12$ and $\alpha =2$, and show a comparison of the numerical results compared to the other existing methods in Table 6, from which one can see that the results of our computations are in good agreement with those ones obtained by the SGM (Babolian et al. 2015), the BSDM (Khuri and Sayfy 2010), the VIM (Wazwaz 2011) and the BSM (Çağlar et al. 2009).

Moreover, since there is no exact solution of this problem, we instead investigate the absolute residual error functions and the maximal error remainder parameters, which are the measures of how well the numerical solution satisfies the original problem (37–38). The absolute residual error functions are

$$\begin{aligned} \left| \hbox {ER}_{N,\alpha }(x)\right| =\left| u_{N,\alpha }^{\prime\prime}(x)+\frac{\alpha }{x} u_{N,\alpha }^{\prime}(x)-\frac{\delta u_{N,\alpha }(x)}{\mu +u_{N,\alpha }(x)}\right| , \quad 0< x \le 1, \end{aligned}$$

and the maximal error remainder parameters are

$$\hbox {MER}_{N,\alpha } = \max \limits _{0< x \le 1} \left| \hbox {ER}_{N,\alpha }(x)\right|.$$

**Fig. 1:**
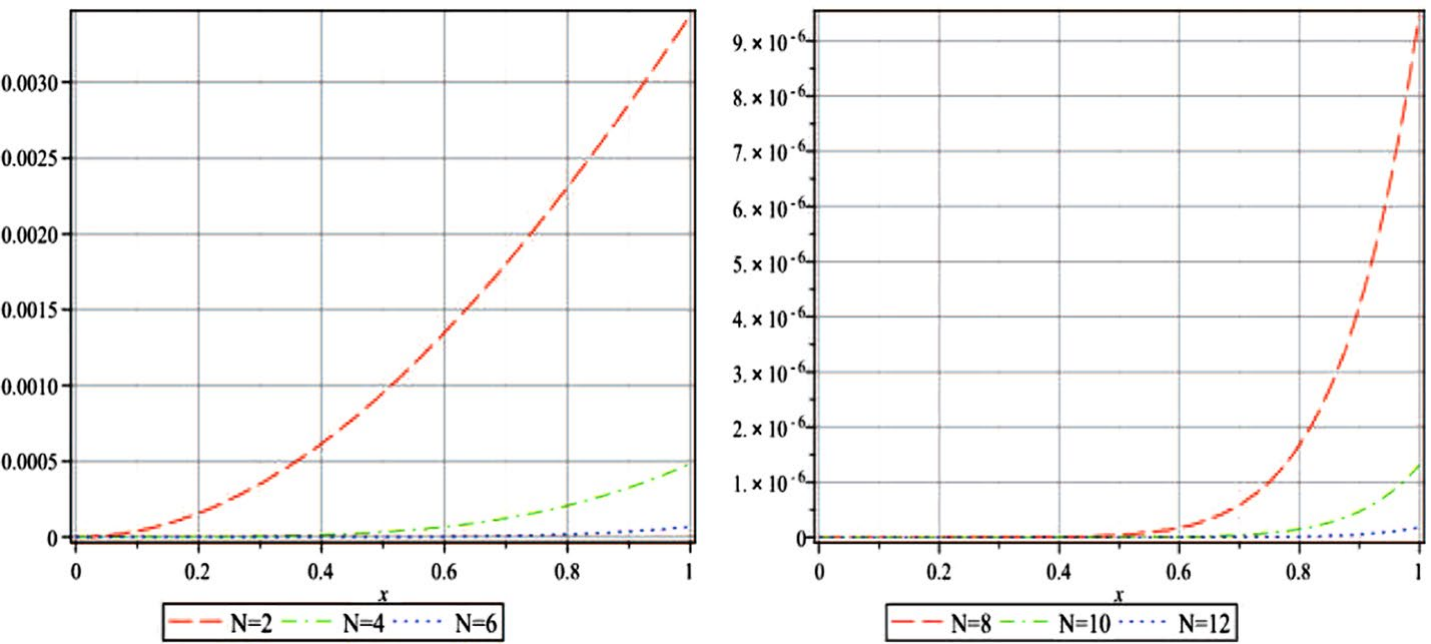
The absolute residual error functions $\left| {\hbox {ER}_{N,2}(x)} \right|$ for $N=2,4,6$ (*left*) and 8, 10, 12 (*right*) of Example 3

**Fig. 2:**
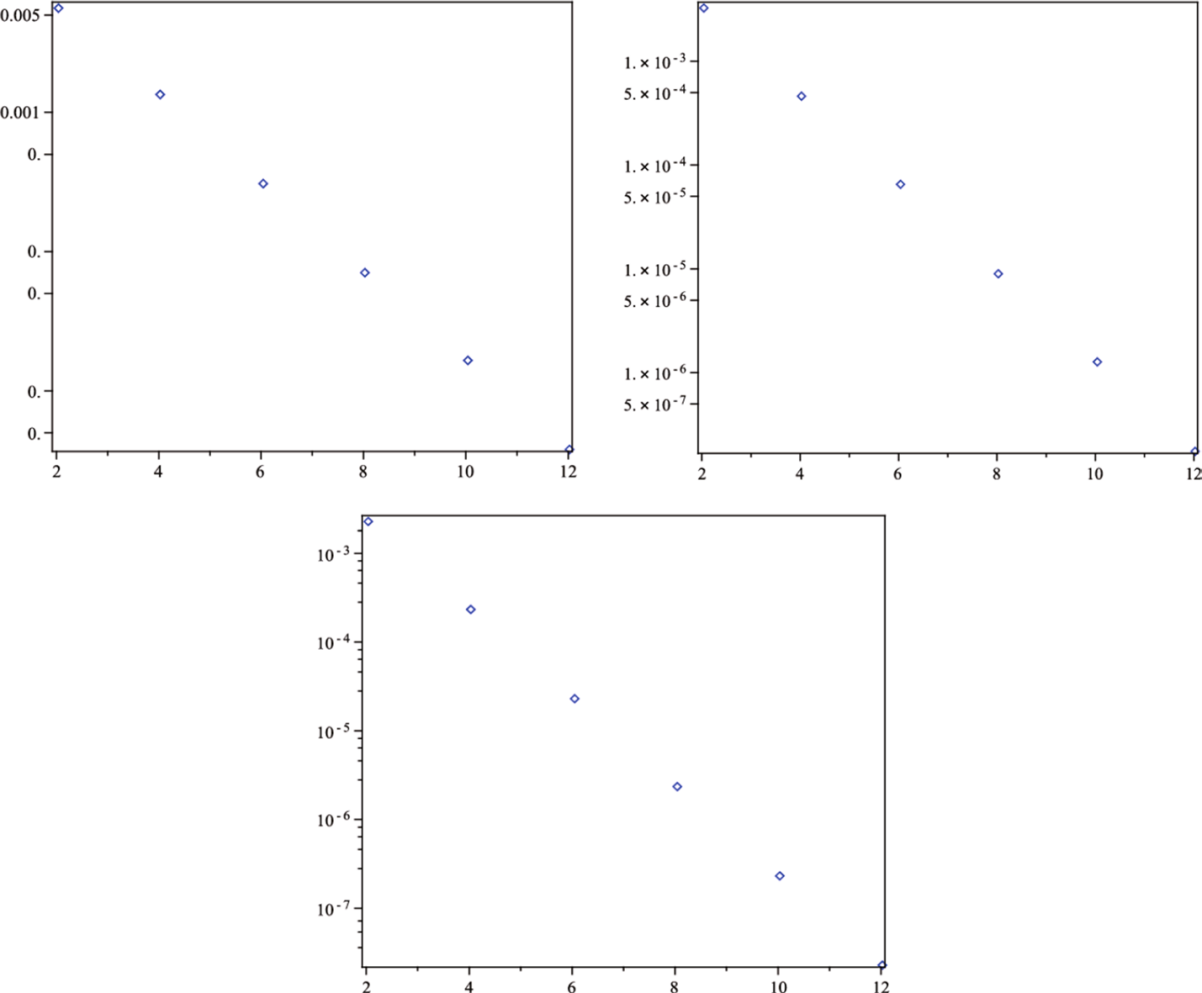
The logarithmic plots for the maximal error remainder parameters $\hbox {MER}_{N,\alpha }$ for $N=2$ through 12 by step 2 and $\alpha =1$ (up, *left*), $\alpha =2$ (up, *right*), $\alpha =3$ (*down*) of Example 3

In Fig. 1, we plot the absolute residual error functions $|\hbox {ER}_{N,2}(x)|$ for $N=2$ through 12 by step 2. Besides, the maximal error remainder parameters $\hbox {MER}_{N,\alpha }$ for the same *N* and $\alpha =1,2,3$ are listed in Table 7, from which it is interesting to point out that for a given *N* the accuracy of our approximate solutions increases with the increase of $\alpha$. Moreover, Fig. 1 and Table 7 show clearly that the accuracy of our method is getting better as the approximation level is increasing for a fixed $\alpha$. The logarithm plots of the value of $\hbox {MER}_{2,\alpha }$ through $\hbox {MER}_{12,\alpha }$ for $\alpha =1,2,3$ are displayed in Fig. 2, which demonstrates an approximately exponential rate of convergence for the obtained truncated series solutions and thus the presented method converges rapidly to the exact solution.

**Table 6:** Comparison of the approximate solutions for Example 3

*x*	BSDM (Khuri and Sayfy 2010)	BSM (Çağlar et al. 2009)	VIM (Wazwaz 2011)	SGM (Babolian et al. 2015)	Present method
0.0	0.8284832948	0.8284832729	0.8284832761	0.8284832912	0.8284832870
0.1	0.8297060968	0.8297060752	0.8297060781	0.8297060933	0.8297060890
0.2	0.8333747380	0.8333747169	0.8333747193	0.8333747345	0.8333747303
0.3	0.8394899183	0.8394898981	0.8394898996	0.8394899148	0.8394899106
0.4	0.8480527887	0.8480527703	0.8480527701	0.8480527859	0.8480527816
0.5	0.8590649275	0.8590649139	0.8590649108	0.8590649281	0.8590649239
0.6	0.8725283156	0.8725283084	0.8725282997	0.8725283208	0.8725283166
0.7	0.8884452994	0.8884452958	0.8884452781	0.8884453065	0.8884453023
0.8	0.9068185417	0.9068185402	0.9068185095	0.9068185490	0.9068185448
0.9	0.9276509830	0.9276509825	0.9276509392	0.9276509893	0.9276509853
1.0	0.9509457948	0.9509457946	0.9509457539	0.9509457994	0.9509457960

**Table 7:** The maximal error remainder parameters $\hbox {MER}_{N,\alpha }$ for Example 3

$\alpha$	$\hbox {MER}_{2,\alpha }$	$\hbox {MER}_{4,\alpha }$	$\hbox {MER}_{6,\alpha }$	$\hbox {MER}_{8,\alpha }$	$\hbox {MER}_{10,\alpha }$	$\hbox {MER}_{12,\alpha }$
1	5.8000e−03	1.4000e−03	3.1751e−04	7.3547e−05	1.7000e−05	3.9243e−06
2	3.4000e−03	4.8431e−04	6.7761e−05	9.4474e−06	1.3142e−06	1.8267e−07
3	2.4000e−03	2.4481e−04	2.4485e−05	2.4388e−06	2.4240e−07	2.4065e−08

### *Example 4*

Consider the following nonlinear SBVP which arises in the study of the distribution of heat sources in the human head (Pandey 1997; Rashidinia et al. 2007; Ravi and Bhattacharya 2006; Babolian et al. 2015; Khuri and Sayfy 2010; Singh and Kumar 2014; Çağlar et al. 2009; Duggan and Goodman 1986):

39 $$u^{\prime\prime}(x)+\frac{2}{x} u^{\prime}(x)=-e^{-u(x)},$$

subject to the boundary conditions

40 $$u^{\prime}(0)=0, \quad au(1)+bu^{\prime}(1)=0.$$

We consider the following two cases:

*Case one: *$$a=b=1.$$

*Case two: *$$a=0.1, b=1.$$

The Adomian polynomials of nonlinear term $f(x,u)=-e^{-u(x)}$ in this problem are computed as

$$\begin{aligned} A_0&= -e^{-U(0)},\\ A_1&= U(1)e^{-U(0)},\\ A_2&= U(2)e^{-U(0)}-\frac{1}{2}U^2(1)e^{-U(0)},\\ A_3&= U(3)e^{-U(0)}-U(1)U(2)e^{-U(0)}+\frac{1}{6}U^3(1)e^{-U(0)},\\&\vdots&\end{aligned}$$

Again no exact solution exists for this equation, hence it was handled numerically. Table 8 describes the numerical results of the first case obtained by the proposed method at the order of approximation $N=12$ and the other existing methods, including the FDM (Pandey 1997), the non-polynomial cubic spline method (NPCSM) (Rashidinia et al. 2007), the CSM (Ravi and Bhattacharya 2006) and the SGM (Babolian et al. 2015). Meanwhile, a comparison for the approximate solutions of the second case obtained by the present method with the same approximation level as the first case and the previous existing methods which include the CSM (Ravi and Bhattacharya 2006), the SGM (Babolian et al. 2015), the BSDM (Khuri and Sayfy 2010) and the BSM (Çağlar et al. 2009) is presented in Table 9. One can seen from two Tables that our computations are in good line with the results obtained by the other approaches compared. In fact, at the approximation level for $N=12$, the maximal absolute error is found to be order of magnitude O($10^{-7}$) for the first case, and O($10^{-9}$) for the second case.

**Table 8:** Comparison of the numerical results for the first case of Example 4

*x*	FDM (Pandey 1997)	NPCSM (Rashidinia et al. 2007)	CSM (Ravi and Bhattacharya 2006)	SGM (Babolian et al. 2015)	Present method
0.0	0.3675169710	0.3675181074	0.3675179806	0.3675168124	0.3675167997
0.1	0.3663623697	0.3663637561	0.3663634922	0.3663623265	0.3663623137
0.2	0.3628941066	0.3628959378	0.3628952219	0.3628940634	0.3628940507
0.3	0.3570975862	0.3570991429	0.3570986892	0.3570975430	0.3570975301
0.4	0.3489484612	0.3489499903	0.3489495462	0.3489484178	0.3489484049
0.5	0.3384121893	0.3384136581	0.3384132502	0.3384121459	0.3384121330
0.6	0.3254435631	0.3254450019	0.3254445925	0.3254435196	0.3254435063
0.7	0.3099860810	0.3099878567	0.3099870705	0.3099860373	0.3099860240
0.8	0.2919711440	0.2919789654	0.2919720836	0.2919711001	0.2919710864
0.9	0.2713170512	0.2713185637	0.2713179289	0.2713170072	0.2713169936
1.0	0.2479277646	0.2479292837	0.2479285659	0.2479277203	0.2479277073

**Table 9:** Comparison of the numerical results for the second case of Example 4

*x*	CSM (Ravi and Bhattacharya 2006)	BSM (Çağlar et al. 2009)	BIDM (Khuri and Sayfy 2010)	SGM (Babolian et al. 2015)	Present method
0.0	1.147041084	1.147039937	1.147040795	1.147039016	1.147039019
0.1	1.146511706	1.146510559	1.146511419	1.146509639	1.146509642
0.2	1.144922563	1.144921418	1.144922282	1.144920499	1.144920502
0.3	1.142270622	1.142269478	1.142270348	1.142268560	1.142268563
0.4	1.138550801	1.138549661	1.138550539	1.138548745	1.138548748
0.5	1.133755950	1.133754813	1.133755703	1.133753900	1.133753904
0.6	1.127876795	1.127875663	1.127876562	1.127874754	1.127874756
0.7	1.120901889	1.120900762	1.120901665	1.120899858	1.120899860
0.8	1.112817535	1.112816416	1.112817317	1.112815517	1.112815520
0.9	1.103607704	1.103606593	1.103607490	1.103605701	1.103605704
1.0	1.093253927	1.093252826	1.093253716	1.093251942	1.093251944

### *Example 5*

Consider the following SBVP with nonlinear term different from the forms (4–7) which arises in the radial stress on a rotationally symmetric shallow membrane cap (Singh and Kumar 2014; Ravi and Aruna 2010):

41 $$\displaystyle u^{\prime\prime}(x)+\frac{3}{x} u^{\prime}(x)=\frac{1}{2}-\frac{1}{8u^2(x)},$$

subject to the boundary conditions

42 $$u^{\prime}(0)=0, \quad u(1)=1.$$

The Adomian polynomials of nonlinear term $f(x,u)=\frac{1}{2}-\frac{1}{8u^2(x)}$ in this problem are computed as

$$\begin{aligned} A_0&= \frac{1}{2}-\frac{1}{8U^2(0)},\\ A_1&= \frac{1}{4} \frac{U(1)}{U^3(0)},\\ A_2&= -\frac{3}{8} \frac{U^2(1)}{U^4(0)}+ \frac{1}{4} \frac{U(2)}{U^3(0)},\\ A_3&= \frac{1}{2} \frac{U^3(1)}{U^5(0)}- \frac{3}{4} \frac{U(1)U(2)}{U^4(0)}+ \frac{1}{4} \frac{U(3)}{U^3(0)},\\&\vdots&\end{aligned}$$

Like the previous problems 3 and 4, a closed-form solution to this equation can not be written down. So we instead investigate the absolute residual error functions and the maximal error remainder parameters to examine the accuracy and the reliability of our numerical results. Here, the absolute residual error functions are

$$\left| \hbox {ER}_N(x) \right| = \left| u_N^{\prime\prime}(x)+\frac{3}{x} u_N^{\prime}(x)-\frac{1}{2}+\frac{1}{8u_N^2(x)} \right| , \quad 0 < x \le 1,$$

and the maximal error remainder parameters are

$$\hbox {MER}_N = \max \limits _{0< x \le 1} \left| \hbox {ER}_N(x) \right|.$$

**Fig. 3:**
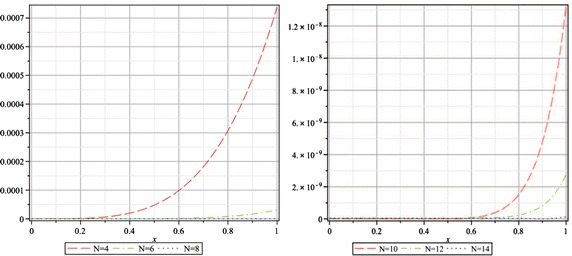
The absolute residual error functions $\left| {\hbox {ER}_N(x)} \right|$ for $N=4,6,8$ (*left*) and 10, 12, 14 (*right*) of Example 5

**Fig. 4:**
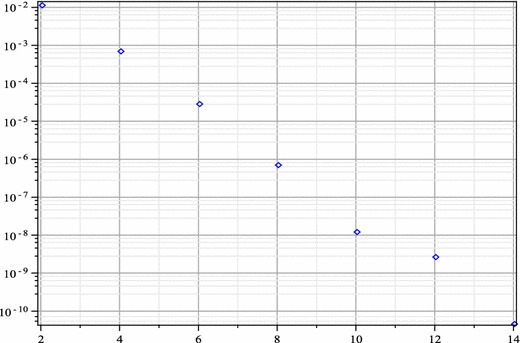
The logarithmic plot for the maximal error remainder parameters $\hbox {MER}_N$ for $N=2$ through 14 by step 2 of Example 5

In Fig. 3, we plot the absolute residual error functions $\left| \hbox {ER}_N(x) \right|$ for $N=4$ through 14 by step 2. The logarithm plot for the maximal error remainder parameters $\hbox {MER}_N$ for the same *N* is shown in Fig. 4, which demonstrates an approximately exponential rate of convergence of the obtained truncated series solutions and thus the presented method converges rapidly to the exact solution. Even though there is no exact solution for this problem, the following 10th order approximation has an accuracy of O($10^{-8}$) and can be used for practical applications

$$\begin{aligned} u_{10}(x)&=0.9541353070+(0.4533672772e-1)x^2+(0.5436871104e-3)x^4-\\&(0.1611538997e-4)x^6+(0.3997114810e-6)x^8-\\&(0.6144814593e-8)x^{10}. \end{aligned}$$

## Conclusion

In this work, a reliable approach based on the IDTM is presented to handle the numerical solutions of a class of nonlinear SBVPs arising in various physical models. This scheme takes the form of a truncated series with easily computable coefficients via the Adomian polynomials of those nonlinearities in the given problem. With the proposed algorithm, there is no need of discretization of the variables, linearization or small perturbation. Numerical results show that the proposed method works well for the SBVPs (1–3) with a satisfying low error. Besides, it is obvious that evaluation of more components of the approximate solution will reasonably improve the accuracy of truncated series solution by using the proposed method. Comparisons of the results reveal that the present method is very effective and accurate. Moreover, we are convinced that the IDTM can be extended to solve the other type of functional equations involving nonlinear terms more easily as the Adomian polynomials are applicable for any analytic nonlinearity and can be generated quickly with the aid of the algorithm proposed by Duan.

It is necessary to point out that algebraic Eq. (23) is a nonlinear one, and we shall inevitably encounter the bad roots while solving it. The criterion to separate the good root from a swarm of bad ones is convergence because it represents the value of unknown function at the origin and will not change for the different *N*.
